# Association of Pretreatment With P2Y12 Receptor Antagonists Preceding Percutaneous Coronary Intervention in Non–ST-Segment Elevation Acute Coronary Syndromes With Outcomes

**DOI:** 10.1001/jamanetworkopen.2020.18735

**Published:** 2020-10-01

**Authors:** Christian Dworeck, Björn Redfors, Oskar Angerås, Inger Haraldsson, Jacob Odenstedt, Dan Ioanes, Petur Petursson, Sebastian Völz, Jonas Persson, Sasha Koul, Dimitrios Venetsanos, Anders Ulvenstam, Robin Hofmann, Jens Jensen, Per Albertsson, Truls Råmunddal, Anders Jeppsson, David Erlinge, Elmir Omerovic

**Affiliations:** 1 Department of Cardiology, Sahlgrenska University Hospital, Gothenburg, Sweden; 2 Department of Cardiology, Danderyd University Hospital, Stockholm, Sweden; 3 Department of Cardiology, Skåne University Hospital, Lund, Sweden; 4 Department of Cardiology, Karolinska University Hospital, Stockholm, Sweden; 5 Department of Cardiology, Östersund Hospital, Östersund, Sweden; 6 Division of Cardiology, Department of Clinical Science and Education, Karolinska Institutet, Södersjukhuset, Stockholm, Sweden; 7 Department of Clinical Science and Education, Karolinska Institutet, Cardiology Capio Sankt Goran Hospital, Stockholm, Sweden; 8 Department of Cardiothoracic Surgery, Sahlgrenska University Hospital, Gothenburg, Sweden

## Abstract

**Importance:**

Pretreatment of patients with non–ST-segment elevation acute coronary syndrome (NSTE-ACS) with P2Y12 receptor antagonists is a common practice despite the lack of definite evidence for its benefit.

**Objective:**

To investigate the association of P2Y12 receptor antagonist pretreatment vs no pretreatment with mortality, stent thrombosis, and in-hospital bleeding in patients with NSTE-ACS undergoing percutaneous coronary intervention (PCI).

**Design, Setting, and Participants:**

This cohort study used prospective data from the Swedish Coronary Angiography and Angioplasty Registry of 64 857 patients who underwent procedures between 2010 and 2018. All patients who underwent PCI owing to NSTE-ACS in Sweden were stratified by whether they were pretreated with P2Y12 receptor antagonists. Associations of pretreatment with P2Y12 receptor antagonists with the risks of adverse outcomes were investigated using instrumental variable analysis and propensity score matching. Data were analyzed from March to June 2019.

**Exposures:**

Pretreatment with P2Y12 receptor antagonists.

**Main Outcomes and Measures:**

The primary end point was all-cause mortality within 30 days. Secondary end points were 1-year mortality, stent thrombosis within 30 days, and in-hospital bleeding.

**Results:**

In total, 64 857 patients (mean [SD] age, 64.7 [10.9] years; 46 809 [72.2%] men) were included. A total of 59 894 patients (92.4%) were pretreated with a P2Y12 receptor antagonist, including 27 867 (43.7%) pretreated with clopidogrel, 34 785 (54.5%) pretreated with ticagrelor, and 1148 (1.8%) pretreated with prasugrel. At 30 days, there were 971 deaths (1.5%) and 101 definite stent thromboses (0.2%) in the full cohort. Pretreatment was not associated with better survival at 30 days (odds ratio [OR], 1.17; 95% CI, 0.66-2.11; *P* = .58), survival at 1 year (OR, 1.34; 95% CI, 0.77-2.34; *P* = .30), or decreased stent thrombosis (OR, 0.81; 95% CI, 0.42-1.55; *P* = .52). However, pretreatment was associated with increased risk of in-hospital bleeding (OR, 1.49; 95% CI, 1.06-2.12; *P* = .02).

**Conclusions and Relevance:**

This cohort study found that pretreatment of patients with NSTE-ACS with P2Y12 receptor antagonists was not associated with improved clinical outcomes but was associated with increased risk of bleeding. These findings support the argument that pretreatment with P2Y12 receptor antagonists should not be routinely used in patients with NSTE-ACS.

## Key Points

**Question:**

Is a pretreatment strategy with P2Y12 receptor antagonists associated with better outcomes vs no pretreatment in patients with non–ST-segment elevation acute coronary syndrome undergoing percutaneous coronary intervention?

**Findings:**

This cohort study including 64 857 patients from the Swedish Coronary Angiography and Angioplasty Registry found that pretreatment with P2Y12 receptor antagonists was not associated with improved survival nor a lower risk of stent thrombosis but was associated with increased risk of bleeding.

**Meaning:**

These findings suggest that pretreatment with P2Y12 receptor antagonists should not be routinely used in non–ST-segment elevation acute coronary syndrome.

## Teaser

This cohort study examines the association of pretreatment with P2Y12 receptor antagonist with 30-day and 1-year mortality and in-hospital bleeding among patients with non–ST-segment elevation acute coronary syndromes (NSTE-ACS).

## Introduction

Early administration of P2Y12 receptor antagonists to patients with non–ST-segment elevation acute coronary syndromes (NSTE-ACS) has been supported by the European Society of Cardiology guidelines for many years^1^ and is a common practice despite the lack of definite evidence for its benefit. However, the American College of Cardiology and American Heart Association do not support pretreatment.^2^ Most of the available data in favor of pretreatment with P2Y12 receptor antagonists in NSTE-ACS are indirect and old.^3,4,5,6,7,8,9^ To our knowledge, no previous trials of the timing of treatment with P2Y12 receptor antagonists have had the adequate statistical power to evaluate mortality and clinically relevant complications. The only randomized clinical trial to our knowledge in patients with NSTE-ACS not only found that pretreatment with a P2Y12 receptor antagonist (ie, prasugrel) was not beneficial but also that pretreatment was harmful, owing to increased risk of major bleeding.^10^ The most recent European Society of Cardiology guidelines from 2015 state, “As the optimal timing of ticagrelor or clopidogrel administration in NSTE-ACS patients scheduled for an invasive strategy has not been adequately investigated, no recommendation for or against pretreatment with these agents can be formulated,” and “based on the ACCOAST results, pretreatment with prasugrel is not recommended.”^11^ In this report based on a nationwide, large prospective cohort of patients with NSTE-ACS undergoing percutaneous coronary intervention (PCI) in Sweden, we compared the associations of P2Y12 receptor antagonists pretreatment vs no pretreatment with mortality, stent thrombosis, and bleeding.

## Methods

The study was approved by the ethical review board in Västra Götaland County. The SWEDEHEART registry from which data were obtained is a nationwide quality registry, and all patients are admitted without the need for consent. However, all patients have the right to be removed from the registry if they wish. Therefore, the review board waived the requirement for patient consent. This study adheres to the Strengthening the Reporting of Observational Studies in Epidemiology (STROBE) reporting guideline for observational studies.

### Databases and Patient Selection

This cohort study used data from the prospective Swedish Coronary Angiography and Angioplasty Registry (SCAAR) database (Figure). Established in 1992, the SCAAR is a national registry of all coronary angiographies and PCIs performed in Sweden. Each catheterization procedure is described with approximately 50 angiographic and 200 PCI variables, including demographic and procedure-related variables. The registry is sponsored by the Swedish Health Authorities and does not receive any funding from commercial interests. All consecutive patients who underwent PCI for NSTE-ACS in Sweden between January 1, 2010, and March 1, 2018, were included in the study. We did not perform sample size analysis before statistical modeling. Patients who did not receive prehospital acetylsalicylic acid or who did not have information about troponin levels before PCI were excluded. More detailed information about SCAAR’s organization and the database has been reported elsewhere.^12,13^ In patients who were hospitalized multiple times during the study period, only the first hospitalization was included in the analysis.

**Figure.**
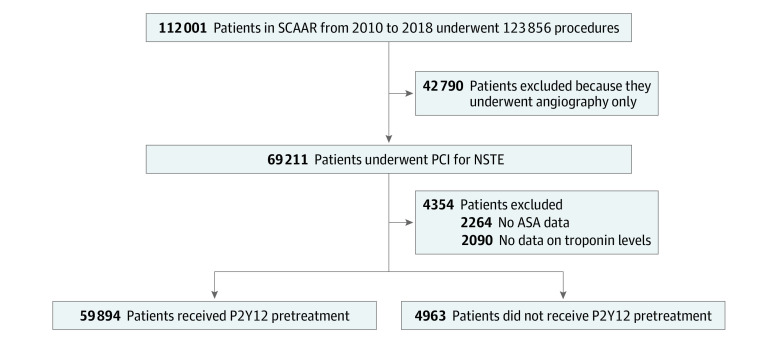
Patient Selection Flowchart Patient data were obtained from the Swedish Coronary Angiography and Angioplasty Registry (SCAAR). ASA indicates acetylsalicylic acid; NSTE, non–ST-segment elevation; PCI, percutaneous coronary intervention.

### Definitions and Outcomes

Patients were considered to have diabetes, hypertension, hyperlipidemia, previous myocardial infarction, or previous stroke if any of those conditions had been diagnosed according to the *International Statistical Classification of Diseases and Related Health Problems, Tenth Revision* (*ICD-10*).^14^ Information about previous PCI, previous coronary artery bypass grafting (CABG), cardiogenic shock, and procedural details are entered directly into the database by interventional cardiologists. Standardized definitions are used across all hospitals for cardiogenic shock and other procedure-related information. Patients who were not Swedish citizens were excluded from the study. Patients were defined as pretreated if they received treatment with clopidogrel, prasugrel, or ticagrelor at any time before coronary angiography. Patients who received P2Y12 antagonists in the catheterization laboratory at the start of PCI were categorized as not pretreated. The treating PCI operator explicitly enters this information into the registry.

#### End Points

The primary end point was mortality within 30 days. Vital status and date of death were obtained from the Swedish National Population Registry until March 14, 2018. The SCAAR was merged with the Swedish National Population Registry by the Epidemiologic Center of the Swedish National Board of Health and Welfare according to Swedish personal identification numbers. Because the use of personal identification numbers is mandatory, the death registry in Sweden has a high degree of completeness, but it is not reviewed or adjudicated to establish cardiac vs noncardiac causes of death.

The secondary end points were stent thrombosis at 30 days and bleeding during the index hospitalization. Stent thrombosis was defined as an acute stent occlusion verified by coronary angiography. In-hospital bleeding was defined as any of the following: puncture site hematoma or pseudoaneurysm, cardiac tamponade, drop in hemoglobin more than 2.0 g/dL (to convert to grams per liter, multiply by 10), prolonged compression treatment, transfusion, or surgical intervention.^15^

#### Prospective Evaluation of the Change in Routine Treatment From Pretreatment to No Pretreatment

During the study period, a change in the clinical routine for pretreatment with P2Y12 receptor antagonists among patients who undergo PCI was made in Västra Götaland county, which accounts for approximately 20% of all observations in the SCAAR. The recommendation not to pretreat patients was issued in April 2016 as part of an administrative decision made by the regional executive board responsible for the organization of health care for patients with ACS. We evaluated the outcomes associated with the change in the policy prospectively by comparing the relevant clinical outcomes before (ie, January 2010 to April 2016) and after (ie, May 2016 to March 2018) the change. In an additional analysis, we evaluated all patients with NSTE-ACS who underwent acute or subacute (during index hospitalization) CABG in Västra Götaland county. We used multivariate logistic regression with the following covariates: age, sex, diabetes, indication for PCI, severity of coronary disease, smoking status, hypertension, hyperlipidemia, previous myocardial infarction, previous PCI, previous CABG, arterial access site, type of stent, type of P2Y12 antagonist, Killip class, completeness of revascularization, and hospital.

### Statistical Analysis

Continuous variables are presented as a median and interquartile range, and categorical variables are presented as numbers and frequencies. Intergroup differences in continuous variables were tested by linear regression. Differences in categorical variables were tested by logistic regression.

#### Statistical Modeling

We used statistical modeling based on the instrumental variable to reduce bias due to unmeasured confounders. This method is a post hoc analytic technique based on statistical principles similar to those used in the analysis of randomized trials (eAppendix in the Supplement).^16,17,18^

Our primary model was based on 2-stage least squares regression^18^ with calendar year as the instrumental variable. The primary outcome variable in the 2-stage least squares regression was mortality at 30 days, and the secondary outcomes were mortality at 1 year, stent thrombosis at 30 days, and in-hospital bleeding. Because the SCAAR is a hierarchical database with clustering of patients within hospitals and regions, we entered individual hospitals into the regression model as random-effects variables. We used multilevel multivariate logistic regression to evaluate the associations of pretreatment with primary and secondary end points of the study in Västra Gätaland county before and after the change in the policy for pretreatment. We assessed trends in 30-day and 1-year mortality over the study period by including the calendar year into the logistic regression as a continuous variable in addition to age and sex.

#### Sensitivity Analyses

In the sensitivity analyses, we used 2-stage least squares regressions with no covariates and propensity score matching. Propensity scores were developed to account for factors associated with being pretreated with a P2Y12 antagonist. Individual propensity scores were calculated through logistic regression modeling based on baseline covariates .^19^ We then matched each patient in the pretreatment group with a patient in the no pretreatment group using 1 to 1 nearest-neighbor matching without replacement (eTable 1 in the Supplement). A standard caliper size of 0.2 × log (SD of the propensity score) was used.^20^

#### Postestimation Diagnostics

Goodness-of-fit (calibration) for the models was assessed with the Hosmer-Lemeshow test. Multicollinearity between the variables in the model was assessed by calculation of the variance inflation factor. All statistical analyses were performed using Stata software version 16.1 (StataCorp). Instrumental variable 2-stage least-squares regression models were completed using the *IVREG2* module. Because of multiple analyses, *P* < .05 was expected to occur accidentally in 1 of 20 analyses. The validity of instrumental variables was examined by calculation of the standardized difference of variables that reflects known patient characteristics and procedural details in treated and untreated patients stratified on the calendar year during the study period (eTable 2 in the Supplement). We used logistic regression to evaluate the estimation power of instruments for pretreatment with P2Y12 antagonists as well as for primary and secondary outcomes. All tests were 2-tailed, and *P* < .05 was considered statistically significant. Data were analyzed from March to June 2019.

## Results

### Patient Characteristics and Treatments

We identified 69 211 patients with NSTE-ACS who underwent PCI during the study period (Figure). We excluded 2264 patients who did not receive acetylsalicylic acid before PCI and 2090 patients with missing data for troponins. The remaining 64 857 patients (mean [SD] age, 64.7 [10.9] years; 46 809 [72.2%] men) were included in the study; 4963 patients (mean [SD] age, 69 [10] years; 3603 [72.6%] men) were not pretreated with a P2Y12 receptor antagonist, and 59 894 patients (mean [SD] age, 68 [10] years; 43 206 [72.1%] men) were pretreated with a P2Y12 receptor antagonist. Patients were reported from 30 different hospitals, and the median (range) of reported patients per hospital was 2009 (427-4066) patients. The proportion of patients who were pretreated decreased by 2.6% (95% CI, 0.8%-4.0%) annually between 2010 and 2018 (*P* < .001) (eFigure 1 in the Supplement).

The characteristics of the patients are presented in Table 1, and procedure-related details are presented in Table 2. Patients pretreated with a P2Y12 receptor antagonist, compared with those who were not, were more likely to be active smokers (11 158 patients [18.6%] vs 738 patients [14.8%]) and less likely to have hypertension (37 869 patients [63.2%] vs 3374 patients [68.0%]), diabetes (13 282 patients [22.2%] vs 1189 patients [23.9%]), myocardial infarction (16 431 patients [27.4%] vs 1328 patients [26.7%]), or prior CABG (5202 patients [8.7%] vs 511 patients [10.3%]). Patients who were pretreated, compared with those who were not, were more likely to have non–ST elevation MI but less likely to develop acute heart failure, lung edema, and cardiogenic shock. During PCI, patients who were pretreated were less likely to have a radial access site and to have PCI performed during off-hours (Table 2). Patients who were pretreated were more often treated with clopidogrel, bivalirudin, and a GP2b/3a receptor antagonist but less often with ticagrelor, prasugrel, and heparin (Table 2). They were more likely to have complex coronary artery disease, complete revascularization during the index PCI, and thrombus aspiration before stent placement but were less likely to receive direct stenting with a drug-eluting stent (Table 2). The utilization of pretreatment with P2Y12 antagonists, outcomes, and patient characteristics stratified by calendar year are presented in eTable 3 in the Supplement.

**Table 1.** Patient Characteristics

Characteristic	No. (%)				*P* value
	Pretreated		Not pretreated		
	Patients (n = 59 894)	Missing	Patients (n = 4963)	Missing	
Age, y					
Mean (SD)	68 (10)	0	69 (10)	0	<.001
>75	16 558 (27.7)	0	1483 (29.9)	0	.001
Men	43 206 (72.1)	0	3603 (72.6)	0	.49
Diabetes	13 282 (22.2)	152 (0.3)	1189 (23.9)	11 (0.2)	.01
Hypertension	37 869 (63.2)	347 (0.6)	3374 (68.0)	39 (0.8)	<.001
Smoking					
Never	23 517 (39.5)	2025 (3.3)	1988 (40.1)	276 (5.6)	<.001
Previous	23 194 (38.7)		1961 (39.5)		
Current	11 158 (18.6)		738 (14.8)		
Hyperlipidemia	31 383 (52.4)	0	2588 (52.1)	0	.35
Previous myocardial infarction	16 431 (27.4)	770 (1.3)	1328 (26.7)	85 (1.7)	.03
Previous PCI	14 839 (24.8)	12 (<0.1)	1182 (23.8)	0	.19
Previous CABG	5202 (8.7)	0	511 (10.3)	1 (<0.1)	.001
Indication for PCI					
Unstable angina	13 212 (22.1)	0	1886 (38.1)	0	<.001
NSTEMI	46 682 (77.9)		3077 (61.9)		
Time to angiography or PCI, mean (SD), d	1.9 (2.4)	0	1.9 (3.4)	0	.901
Killip class					
I	44 476 (96.5)	13 819 (23.1)	3930 (94.9)	823 (16.6)	<.001
II	1014 (2.2)		131 (3.2)		
III	289 (0.6)		38 (0.9)		
IV	134 (0.3)		36 (0.9)		

Abbreviations: CABG, coronary artery bypass grafting; NSTEMI, non–ST segment elevation myocardial infarction; PCI, percutaneous coronary intervention.

**Table 2.** Angiography and PCI

Intervention	No. (%)				*P* value
	Pretreated		Not pretreated		
	Patients (n = 59 894)	Missing	Patients (n = 4963)	Missing	
Radial artery access	47077 (78.6)	640 (1.1)	4070 (81.8)	63 (1.3)	<.001
Procedure performed off-hours	9552 (15.9)	663 (1.1)	1012 (20.4)	9 (0.2)	<.001
Arteries with stenosis					
0	2334 (3.9)	115 (0.3)	305 (6.2)	46 (0.7)	<.001
1	27 089 (45.2)		2138 (43.1)		
>2 and/or LM	30 471 (50.9)		2520 (50.8)		
Complete revascularization	37 483 (62.6)	5273 (0.9)	2923 (58.9)	716 (1.3)	<.001
PCI with stent					
Drug-eluting stent	42 813 (71.8)	0	3849 (77.6)	0	<.001
Bare metal stent	8214 (13.7)		206 (4.2)		
No stent	8867 (14.8)		908 (18.3)		
P2Y12 receptor antagonist					
Clopidogrel	27 129 (45.3)	0	738 (18.9)	1057 (21.3)	<.001
Ticagrelor	31 706 (52.9)		3079 (78.8)		
Prasugrel	1059 (1.8)		89 (2.3)		
Thrombus aspiration	1351 (2.3)	67 (0.2)	42 (0.9)	44 (0.6)	<.001
Direct stenting	8874 (14.8)	2 (0.0)	880 (17.8)	0	<.001
Bivalirudin	9544 (15.9)	492 (1.3)	436 (8.8)	1 (0.1)	<.001
GP2b/3a receptor inhibitor	1555 (2.6)	1 (0.0)	97 (1.9)	1 (0.1)	.002
Unfractionated heparin	53 338 (89.1)	4 (0.1)	4485 (90.4)	1 (0.1)	.007

Abbreviations: LM, left main; PCI, percutaneous coronary intervention.

### Clinical Outcomes

#### Primary End Point

Between 2000 and 2018, the overall mortality at 30 days did not change (unadjusted odds ratio [OR], 0.99; 95% CI, 0.97-1.01; *P* for trend = .77). At 30 days, there were 971 deaths (1.5%) and 101 definite stent thromboses (0.2%). After adjustment for age and sex only, mortality at 30 days was lower in patients who were pretreated compared with those who were not (846 deaths [1.4%] vs 125 deaths [2.5%]; OR, 0.54; 95% CI, 0.45-0.66; *P* < .001). The primary model, based on the instrumental variable analysis with additional covariates, showed no difference in 30-day mortality between the groups (OR, 1.44; 95% CI, 0.78-2.62; *P* = .36) (Table 3). We found no evidence for effect modification by type of P2Y12 antagonist (interaction test, *P* = .16) and unstable angina or NSTEMI (interaction test, *P* = .12) on the primary end point.

**Table 3.** Primary Analysis

Clinical outcome	Patients, No. (%)			Adjusted OR (95% CI)	*P* value
	Pretreated (n = 59 894)	Not pretreated (n = 4963)	Missing		
Primary end point					
Death at 30 d^a^^,^^b^	846 (1.4)	125 (2.5)	0	1.44 (0.78-2.62)	.36
Secondary end point					
Death at 1 y^a^^,^^c^	2324 (4.3)	241 (7.1)	0	1.34 (0.77-2.34)	.30
Definite stent thrombosis at 30 d^a^^,^^d^	243 (0.2)	19 (0.2)	0	1.17 (0.64-2.16)	.60
In-hospital bleeding^a^^,^^e^	3562 (6.0)	380 (7.5)	11 (0.1)	1.49 (1.06-2.12)	.02

Abbreviation: OR, odds ratio.

^a^ Instrumental variable analysis. The following variables were entered into regression in addition to the instrumental variable: age, sex, diabetes, indication for percutaneous coronary intervention, the severity of the coronary disease, smoking status, hypertension, hyperlipidemia, previous myocardial infarction, previous percutaneous coronary intervention, previous coronary artery bypass graft, arterial access site, type of stent, type of P2Y12 antagonist, Killip class, completeness of revascularization, and hospital.

^b^ Underidentification test, *P* = .02; weak identification test (*F* statistic), 357; overidentification test of all instruments, *P* = .89.

^c^ Underidentification test, *P* = .006; weak identification test (*F* statistic), 275; overidentification test of all instruments, *P* = .51.

^d^ Underidentification test, *P* = .01; weak identification test (*F* statistics), 356; overidentification test of all instruments, *P* = .77.

^e^ Includes major bleeding (Bleeding Academic Research Consortium type 3) and minor bleeding (Bleeding Academic Research Consortium type 2). Underidentification test, *P* = .02; weak identification test (*F* statistic), 372; overidentification test of all instruments, *P* = .61.

#### Secondary End Points

Between 2000 and 2018, the overall mortality at 1 year did not change (OR, 0.99; 95% CI, 0.98-1.02; *P* = .79). After adjustment for age and sex, mortality at 1 year was lower in patients who were pretreated compared with those who were not (2324 deaths [4.3%] vs 241 deaths [7.1%]; OR, 0.63; 95% CI, 0.54-0.72; *P* < .001). However, the instrumental variable analysis showed no difference in 1-year mortality between the groups (adjusted OR, 1.34; 95% CI, 0.77-2.34; *P* = .30) (Table 3). We found no evidence for effect modification by type of P2Y12 antagonist (interaction test, *P* = .24) and unstable angina or NSTEMI (interaction test, *P* = .17) on the secondary end point of 1-year mortality.

#### Stent Thrombosis

The overall incidence of definite stent thrombosis at 30 days was 262 patients (0.2%); unadjusted and adjusted risk was not different in patients with vs without P2Y12 receptor antagonist pretreatment (243 patients [0.2%] vs 19 patients [0.2%]; unadjusted OR, 1.09; 95% CI, 0.68-1.74; *P* = .71; adjusted OR, 1.17; 95% CI, 0.64-2.16; *P* = .60) (Table 3). We found no evidence for effect modification by type of P2Y12 antagonist (interaction test, *P* = .37) and unstable angina or NSTEMI (interaction test, *P* = .54) on the secondary end point of stent thrombosis.

#### In-Hospital Bleeding

Data on bleeding were missing in 11 patients (0.1%). Bleeding occurred in 3956 patients (6.1%). The incidence of bleeding was lower in patients with P2Y12 receptor antagonist pretreatment (3562 patients [6.0%] vs 380 patients [7.5%]; unadjusted OR, 0.76; 95% CI, 0.68-0.84; *P* < .001), but after the adjustment, the risk estimate was higher in the patients who were pretreated (adjusted OR 1.49; 95% CI, 1.06-2.12; *P* = .02) (Table 3). In-hospital bleeding was associated with a substantially higher risk of death at 30 days (adjusted OR, 8.68; 95% CI, 7.54-9.98; *P* < .001) and at 1 year (adjusted OR, 3.05; 95% CI, 2.73-3.42; *P* < .001). After the exclusion of minor bleeding complications (ie, hematomas and pseudoaneurysms), the risk of bleeding was still higher with P2Y12 receptor antagonist pretreatment (adjusted OR, 2.31; 95% CI, 1.34-3.98; *P* = .002). We found no evidence for effect modification by type of P2Y12 antagonist (interaction test, *P* = .34) and unstable angina or NSTEMI (interaction test, *P* = .64) on the secondary end point of in-hospital bleeding.

#### Prospective Evaluation of the Change in Routine Treatment From Pretreatment to No Pretreatment

This prespecified prospective evaluation was performed in 13 720 patients, of whom 3655 (27.8%) were not pretreated. After the change in the practice of pretreatment with P2Y12 receptor antagonists in Västra Götaland county, the number of patients with NSTE-ACS who were pretreated decreased from 894 of 900 patients (99.3%) in 2010 to 48 of 329 patients (14.6%) in 2018 (*P* < .001) (eFigure 2 in the Supplement). We found no difference between the 2 periods in death at 30 days (adjusted OR, 1.15; 95% CI, 0.83-1.59; *P* = .39), death at 1 year (adjusted OR, 1.01; 95% CI, 0.79-1.27; *P* = .96), and definite stent thrombosis at 30 days (adjusted OR, 0.79; 95% CI, 0.42-1.55; *P* = .52) (Table 4). However, the risk of bleeding decreased after April 2016, when patients were not routinely pretreated (adjusted OR, 0.80; 95% CI, 0.69-0.94; *P* = .006).

**Table 4.** Prospective Evaluation of the Change in the Policy for Routine Pretreatment With P2Y12 Receptor Antagonists in Patients With NSTE-ACS in Västra Götaland County Treated With PCI or CABG

Outcome	Patients, No. (%)		Adjusted OR (95% CI)	*P* value
	Routine pretreatment	No routine pretreatment		
PCI, No.	10 065	3655	NA	NA
Death at 30 d^a^	194 (1.9)	81 (2.2)	1.15 (0.83-1.59)	.39
Death at 1 y^a^	545 (5.4)	120 (5.9)	1.01 (0.79-1.27)	.96
Definite stent thrombosis at 30 d^b^	20 (0.2)	5 (0.1)	0.79 (0.42-1.55)	.52
In-hospital bleeding^a^^,^^c^	869 (8.5)	314 (8.1)	0.80 (0.69-0.94)	.006
CABG, No.	1106	724	NA	NA
Death at 30 d^d^	30 (2.7)	14 (1.9)	0.79 (0.41-1.51)	.47
Death at 1 y^d^	55 (4.9)	28 (3.8)	0.85 (0.53-2.34)	.52
Reoperation owing to bleeding^d^^,^^e^	30 (2.7)	14 (1.9)	0.67 (0.41-0.96)	.04

Abbreviations: CABG, coronary bypass surgery; NA, not applicable; NSTE-ACS, non–ST-segment elevation acute coronary syndromes; OR, odds ratio; PCI, percutaneous coronary intervention.

^a^ Logistic regression adjusted for age, sex, diabetes, indication for PCI, severity of coronary disease, smoking status, hypertension, hyperlipidemia, previous myocardial infarction, previous PCI, previous CABG, arterial access site, type of stent, type of P2Y12 antagonists, Killip class, completeness of revascularization, and hospital.

^b^ Multilevel logistic regression adjusted for age, sex, diabetes, indication for PCI, severity of coronary disease, smoking status, hypertension, hyperlipidemia, previous myocardial infarction, previous PCI, previous CABG, arterial access site, type of stent, stent length, stent diameter, type of P2Y12 antagonists, Killip class, completeness of revascularization, and hospital.

^c^ Includes major bleeding (Bleeding Academic Research Consortium type 3) and minor bleeding (Bleeding Academic Research Consortium type 2).

^d^ Logistic regression adjusted for Euroscore II.

^e^ Bleeding Academic Research Consortium type 4.

In total, 1830 patients underwent CABG, of whom 724 (39.6%) were treated after April 2016 and hence did not receive pretreatment with P2Y12 receptor antagonists. We found no difference in patients who underwent CABG due to NSTE-ACS during index hospitalization between the 2 periods in death at 30 days (adjusted OR, 0.79; 95% CI, 0.41-1.51; *P* = .47) or at 1 year (adjusted OR, 0.85; 95% CI, 0.53-2.34; *P* = .52) (Table 4). However, the risk for reoperation owing to bleeding was substantially lower in patients during the second period (ie, after routine pretreatment was halted) (adjusted OR, 0.67; 95% CI, 0.41-0.96; *P* = .04).

### Sensitivity Analysis and Postestimation Diagnostics

The results from the sensitivity analyses were congruent with the results from the primary model (eTable 1 and eTable 2 in the Supplement). Postestimation analysis for the logistic regression models by Hosmer-Lemeshow test showed adequate goodness of fit for the models. Squared covariate terms had no explanatory power in any of the models. The mean variance inflation factor was less than 5.0 for all models, indicating a lack of multicollinearity between the variables.

## Discussion

This cohort study among 64 8857 patients undergoing PCI for NSTE-ACS in Sweden between January 2010 and March 2018 found that pretreatment with P2Y12 receptor antagonists was not associated with improved survival or with a lower likelihood of definite stent thrombosis. Furthermore, we found that pretreatment with P2Y12 receptor antagonists was associated with a higher risk of bleeding and that the change in the practice from routine pretreatment to no pretreatment was associated with decreased risk of bleeding.

Anticipated benefits of pretreatment with P2Y12 receptor antagonists before PCI include a reduction in ischemic events, the potential to reduce PCI complications (eg, acute stent thrombosis), and a reduced need for more potent glycoprotein IIb/IIIa receptor inhibitors. As such, the administration of P2Y12 receptor antagonists is often started as soon as NSTE-ACS is clinically suspected. Potential downsides of pretreatment include an increased risk of bleeding, especially if these agents are administered mistakenly to patients with contraindications (eg, aortic dissection, ongoing bleeding, subarachnoid hemorrhage). Additionally, there is a higher risk of CABG-associated bleeding if urgent surgical treatment is required or more extended hospital stays for patients who develop bleeding complications. Whereas the American College of Cardiology and American Heart Association guidelines give no recommendation for pretreatment in NSTE-ACS,^2^ the European Society of Cardiology guidelines stated until 2015 a clear preference for administering P2Y12 antagonists before PCI.^1^ This recommendation was adopted and implemented by Swedish hospitals, which is reflected in this study by the fact that more than 90% patients were pretreated with P2Y12 receptor antagonists.

To our knowledge, the ACCOAST trial^10^ is the only multicenter randomized clinical trial that has directly addressed whether pretreatment in patients with NSTE-ACS is superior to the start of the treatment with P2Y12 receptor antagonist in the PCI theater when the decision has been made to perform PCI after diagnostic angiography. In the ACCOAST trial,^10^ pretreatment with prasugrel was not superior to in-hospital administration regarding the primary end point of cardiovascular death, stroke, myocardial infarction, and urgent revascularization, although bleeding was nearly 2-fold higher in the pretreatment group. In the ISAR-REACT 5 trial,^21^ pretreatment strategy with ticagrelor was inferior to prasugrel which was started after coronary angiography. Our study confirms these findings. Thus, neither the ACCOAST trial^10^ nor our study—among the largest cohort studies to date, to our knowledge—found any beneficial associations of pretreatment with P2Y12 receptor antagonists in NSTE-ACS.

Although our study provides robust external validation of the results from the smaller ACCOAST trial,^10^ the reason for lack of beneficial effect of pretreatment with P2Y12 receptor antagonists on ischemic events as well as the increased risk of bleeding remains to be established. One of the proposed explanations for the lack of effect of pretreatment with P2Y12 administration in the ACCOAST trial^10^ was the relatively short median time from first medical contact to the start of PCI (approximately 4 hours), which could translate to suboptimal platelet inhibition at the time of PCI. However, it has been shown that the level of platelet inhibition was substantial (approximately 30% of the control group) at the time of PCI. Furthermore, subgroup analysis from ACCOAST trial^10^ found that pretreatment of patients with longer delay to PCI (ie, >14 hours) was not associated with lower rates of ischemic events. Nevertheless, whether pretreatment with P2Y12 antagonists to selected patients can improve clinical outcomes remains to be established. An alternative explanation for the lack of benefit with the pretreatment strategy is that this therapy is futile under the current circumstance with shorter time to PCI, use of more potent antiplatelet agents during and after PCI, and improved technology and operators’ skills. Therefore, pretreatment may be associated with more harm than benefit in the modern clinical era.

The number of patients who are initially mistakenly diagnosed with NSTE-ACS in everyday clinical practice is substantial. Even in carefully designed and conducted clinical trials, the risk of diagnostic errors is surprisingly high. In the ACUITY study,^22^ after careful assessment, approximately 30% of all included patients turned out not to have NSTE-ACS, which was the key inclusion criterion. Indeed, some observational studies suggest that the number of inappropriate initiations of antiplatelet therapy in patients with chest pain and aortic dissection in Sweden may be as high as 60%.^23^ These facts create an obvious ethical problem because futile treatment, which causes substantial harm, should not be recommended nor used. This is particularly important because the incidence of NSTE-ACS in the general population is high. Since accidental treatment with P2Y12 receptor antagonists in patients with contraindications is a well-known problem, had these patients been included in the ACCOAST trial,^10^ the harmful effects would most likely have been greater.

### Limitations

Our observational study has several limitations. We cannot exclude selection bias and residual confounding. Nevertheless, our study provides real-world data from a large cohort of patients. Our prospective evaluation of relevant health care outcomes after the change in the clinical practice provides evidence for an association between pretreatment and increased risk of bleeding in patients undergoing PCI and CABG. The study is based on the data from a nationwide registry with complete coverage of all patients with NSTE-ACS who underwent PCI in Sweden. During the study period, we have conducted several large randomized registry clinical trials (TASTE,^13^ DETO2X-SWEDEHEART,^24^ iFR-SWEDEHEART,^25^ and VALIDATE-SWEDEHEART^26^) within the registry platform. We do not have data on cause-specific mortality. We did not include patients mistakenly diagnosed with NSTE-ACS and who were not treated with PCI, because pretreatment in these patients is not reported in the registry. Previous studies have reported that inadvertent prehospital administration of antiplatelet drugs to patients with contraindications to antithrombotic therapy is frequent. Therefore, including such patients in the analyses could have influenced the risk estimates toward an even higher increased risk of adverse cardiovascular events with pretreatment. The SCAAR does not gather information about patients who die before hospitalization, and we were unable to provide these data. We did not correct for multiple testing. We did not adjust for the switch in P2Y12 antagonists, which is known to occur among a portion of patients treated with PCI.^27^ We did not have information about treatment with P2Y12 antagonists before the hospital admission. Some factors related to the patient's characteristics, adjunctive treatment regimens, and logistics in health care services changed throughout the study. However, we accounted for these changes in clinical practice by using the calendar year as a treatment-preference instrument in the statistical models. The number of events per variable for some end points in the primary model was low, which has lowered statistical power and may have increased the risk of bias. However, the results from the primary analysis and all the sensitivity analyses were congruent, which speaks against the presence of significant bias.

## Conclusions

This cohort study among patients undergoing PCI for NSTE-ACS in Sweden between 2010 and 2018 found that pretreatment with P2Y12 receptor antagonists was not associated with improved survival at 30 days and 1 year but rather with substantially increased risk of in-hospital bleeding. Our findings independently validate the results of the multicenter randomized ACCOAST^10^ and ISAR-REACT 5 trials.^21^

## Notes

Supplement. **eAppendix.** Description of Instrumental Variable Analysis **eTable 1.** Sensitivity Analysis With 1:1 Propensity Score Matching **eTable 2.** Instrumental Variable Analysis Without Covariates **eTable 3.** Utilization of Pretreatment With P2Y12 Antagonists, Outcomes, and Patient Characteristics Stratified by Calendar Year **eFigure 1.** Changing Trend in Pretreatment With P2Y12 Receptor Antagonists Before PCI in Patients With NSTE-ACS Between 2010 and 2018 in Sweden **eFigure 2.** Frequency in Pretreatment With P2Y12 Receptor Antagonists Before and After the Change in the Policy for Routine Pretreatment With P2Y12 Receptor Antagonists Before PCI in Patients With NSTE-ACS in Västra Götaland County
