# Supplementary material for: Association of Pretreatment With P2Y12 Receptor Antagonists Preceding Percutaneous Coronary Intervention in Non–ST-Segment Elevation Acute Coronary Syndromes With Outcomes
Source: JAMA Netw Open. 2020 Oct 1;3(10):e2018735. doi: 10.1001/jamanetworkopen.2020.18735 (PMC7530628; doi:10.1001/jamanetworkopen.2020.18735)
Supplement: Supplement. — eAppendix. Description of Instrumental Variable Analysis eTable 1. Sensitivity Analysis With 1:1 Propensity Score Matching eTable 2. Instrumental Variable Analysis Without Covariates eTable 3. Utilization of Pretreatment With P2Y12 Antagonists, Outcomes, and Patient Characteristics Stratified by Calendar Year eFigure 1. Changing Trend in Pretreatment With P2Y12 Receptor Antagonists Before PCI in Patients With NSTE-ACS Between 2010 and 2018 in Sweden eFigure 2. Frequency in Pretreatment With P2Y12 Receptor Antagonists Before and After the Change in the Policy for Routine Pretreatment With P2Y12 Receptor Antagonists Before PCI in Patients With NSTE-ACS in Västra Götaland County [file jamanetwopen-e2018735-s001.pdf]

Supplemental Online Content

Dworeck C, Redfors B, Angerås O, et al. Association of pretreatment with P2Y12 receptor antagonists preceding percutaneous coronary intervention in non–ST-segment elevation acute coronary syndromes with outcomes. *JAMA Netw Open*. 2020;3(10):e2018735. doi:10.1001/jamanetworkopen.2020.18735

eAppendix. Description of Instrumental Variable Analysis

eTable 1. Sensitivity Analysis With 1:1 Propensity Score Matching

eTable 2. Instrumental Variable Analysis Without Covariates

eTable 3. Utilization of Pretreatment With P2Y12 Antagonists, Outcomes, and Patient Characteristics Stratified by Calendar Year

eFigure 1. Changing Trend in Pretreatment With P2Y12 Receptor Antagonists Before PCI in Patients With NSTE-ACS Between 2010 and 2018 in Sweden

eFigure 2. Frequency in Pretreatment With P2Y12 Receptor Antagonists Before and After the Change in the Policy for Routine Pretreatment With P2Y12 Receptor Antagonists Before PCI in Patients With NSTE-ACS in Västra Götaland County

This supplemental material has been provided by the authors to give readers additional information about their work.

eAppendix. Description of Instrumental Variable Analysis

We based our primary statistical model on the instrumental variable analysis to reduce bias due to unmeasured and unknown confounders. This method is a post hoc analytic technique based on statistical principles similar to those used in the analysis of randomized controlled trials[1-3]. To use instrumental variable analysis, one must identify a naturally varying phenomenon in the observed data, which like the act of randomization in an RCT, predicts the treatment that will be assigned to the individual patient. To become a valid instrument, a variable must fulfill some necessary criteria. First, it must be strongly associated with the received treatment. Second, it must not be associated directly or indirectly with the outcome, except through the effect of the treatment itself. The variable with these statistical qualities is called instrumental variable, or instrument. We used the calendar year as the treatment-preference instruments. Calendar time is frequently employed as instruments because this type of variables usually fulfills the theoretical criteria for a valid instrument[4-6]. Variations in the use of the pretreatment strategy over time in Sweden is a result of changes in guidelines and reimbursement policies as well as changes in physicians' preference due to the release of new effectiveness and safety information. Durbin-Wu-Hausman specification test was used to evaluate the presence of residual confounding (endogeneity). The validity of the instrumental variable was tested with the Sargan test. To test for the strength of the instruments, we examined the partial F test from the first-stage regression, which predicts treatment as a function of instrument and covariates. The partial F test has the null hypothesis that the coefficient for the effect of the instrument in the first-stage regression model is zero[7]. An F-statistic greater than 10 indicates that the instrument is not weak. Reported standard errors from IV 2SLS regression are robust and account for clustering of patients within hospitals using the sandwich estimator. An imperfect instrument may become valid after conditioning on an adequately chosen set of auxiliary variables[8]. Because "calendar year" may be an imperfect instrument, the following variables were entered into IV regression: age, sex, diabetes, indication for PCI, the severity of the coronary disease, smoking status, hypertension, hyperlipidemia,

© 2020 Dworeck C et al. *JAMA Network Open.*

previous myocardial infarction, previous PCI, previous coronary artery bypass graft, arterial access site, type of stent, type of $P2Y_{12}$ antagonists, Killip class, completeness of revascularization and hospital.

Durbin-Wu-Hausman specification test was used to evaluate the presence of residual confounding (endogeneity). The validity of the instrumental variable was tested with the Sargan test. To test for the strength of the instruments, we examined the partial F test from the first-stage regression, which predicts treatment as a function of instrument and covariates. The partial F test has the null hypothesis that the coefficient for the effect of the instrument in the first-stage regression model is zero[7]. An F-statistic greater than 10 indicates that the instrument is not weak. Reported standard errors from IV 2SLS regression are robust and account for clustering of patients within hospitals using the sandwich estimator. Our primary model was based on instrumental variable two-stage least squares (2SLS) regression[3]. The outcome (dependent) variables in the 2SLS regressions were all-cause mortality at 30-days or definite stent thrombosis at 30-days, all-cause mortality 30-days, all-cause mortality at one-year, in-hospital bleeding, in-hospital neurologic complications.

References

1. Harris KM, Remler DK. Who is the marginal patient? Understanding instrumental variables estimates of treatment effects. Health Serv Res 1998;33:1337-60.
2. Mcclellan M, Mcneil BJ, Newhouse JP. Does More Intensive Treatment of Acute Myocardial-Infarction in the Elderly Reduce Mortality - Analysis Using Instrumental Variables. Jama-J Am Med Assoc 1994;272:859-66.
3. Rassen JA, Schneeweiss S, Glynn RJ, Mittleman MA, Brookhart MA. Instrumental Variable Analysis for Estimation of Treatment Effects With Dichotomous Outcomes. Am J Epidemiol 2009;169:273-84.
4. Stukel TA, Fisher ES, Wennberg DE, Alter DA, Gottlieb DJ, Vermeulen MJ. Analysis of observational studies in the presence of treatment selection bias: effects of invasive cardiac management on AMI survival using propensity score and instrumental variable methods. JAMA 2007;297:278-85.
5. Brookhart MA, Rassen JA, Schneeweiss S. Instrumental variable methods in comparative safety and effectiveness research. Pharmacoepidemiol Drug Saf 2010;19:537-54.
6. Earle CC, Tsai JS, Gelber RD, Weinstein MC, Neumann PJ, Weeks JC. Effectiveness of chemotherapy for advanced lung cancer in the elderly: Instrumental variable and propensity analysis. J Clin Oncol 2001;19:1064-70.

© 2020 Dworeck C et al. *JAMA Network Open.*

7. Bound J, Jaeger DA, Baker RM. Problems with Instrumental Variables Estimation When the Correlation Between the Instruments and the Endogeneous Explanatory Variable is Weak. Journal of the American Statistical Association 1995;90:443-50.
8. Brito C, Pearl J. Generalized Instrumental Variables. UAI'02: Uncertainty in Artificial Intelligence, Proceedings of the Eighteenth Conference: Morgan Kaufmann Publishers Inc., 340 Pine Street, Sixth Floor, San Francisco, CA, United States; 2002:85–93.

© 2020 Dworeck C et al. *JAMA Network Open.*

eTable 1. Sensitivity Analysis With 1:1 Propensity Score Matching

**Clinical outcome**	**Pretreated (N= 3,481)**	**Not pretreated (N= 3,481)**	**Adjusted OR**	**95% CI**	**P-value**	**Missing n (%)**	
**Primary endpoint:**							
Death at 30 days — no. (%)*	103 (3.0)	81 (2.3)	1.28	0.95-1.72	0.100	0	
**Secondary endpoints:**							
Death at one year — no. (%)*	179 (7.1)	193 (7.6)	1.01	0.68-1.48	0.968	0	
Definite stent thrombosis at 30 days — no. (%)*	13 (0.4)	6 (0.2)	1.41	0.44-4.44	0.562	0	
In-hospital bleeding— no. (%)*#	23 (0.6)	23 (0.6)	1.19	1.01-1.41	0.033	0	

* Propensity score matching 1:1.
OR=odds ratio
[#] major bleeding (BARC type 3), minor bleeding (BARC type 2)

© 2020 Dworeck C et al. *JAMA Network Open.*

eTable 2. Instrumental Variable Analysis Without Covariates

Clinical outcome	Pretreated (N= 59,894)	Not pretreated (N= 4,963)	Adjusted OR	95% CI	P-value	Missing n (%)	
**Primary endpoint:**							
Death at 30 days — no. (%)	846 (1.4)	125 (2.5)	1.17	0.66-2.09	0.594	0	
**Secondary endpoints:**							
Death at one year — no. (%)*	2,324 (4.3)	241 (7.1)	0.96	0.56-1.63	0.879	0	
Definite stent thrombosis at 30 days — no. (%)*	243 (0.2)	19 (0.2)	2.79	0.59-13.3	0.196	0	
In-hospital bleeding— no. (%)*#	3,562 (6.0)	380 (7.5)	1.41	1.01-2.01	0.048	0	

OR odds ratio
major bleeding (BARC type 3), minor bleeding (BARC type 2),

© 2020 Dworeck C et al. *JAMA Network Open.*

eTable 3. Utilization of Pretreatment With P2Y12 Antagonists, Outcomes, and Patient Characteristics Stratified by Calendar Year

	2010 (N =6,933)	2011 (N =7,462)	2012 (N =8,200)	2013 (N =7,894)	2014 (N =8,325)	2015 (N =8,146)	2016 (N =8,249)	2017 (N =8,537)	2018 (N =1,111)	Stand. Diff.
Pretreated with $P2Y_{12}$ (%)	100	97.8	95.9	95.7	95.8	94.0	83.5	80.5	79.2	
Death at 30 day (%)	1.7	1.3	1.5	1.5	1.5	1.6	1.5	1.5	1.3	0.02
Death at one year (%)	5.0	4.3	4.3	4.5	4.4	4.6	4.7	4.0		0.05
Definite stent thrombosis at 30 days (%)	0.2	0.2	0.2	0.2	0.2	0.2	0.1	0.1	0.3	0.01
In-hospital bleeding (%)	6.8	5.8	6.6	6.4	6.1	6.0	6.2	6.1	4.8	0.09
Age (mean±SD)	68±11	68±11	68±11	68±11	68±11	68±11	69±11	69±11	69±11	0.08
Age ≥75 (%)	29.6	29.9	28.3	29.6	30.7	30.3	31.1	31.7	32.7	0.04
Female sex (%)	27.8	27.4	27.2	26.5	27.5	28.1	27.3	27.9	29.1	0.05
Diabetes (%)	21.6	20.9	20.1	21.4	21.8	22.5	21.5	23.7	25.0	0.02
Hypertension (%)	59.8	61.8	63.0	63.5	63.8	63.3	64.5	65.6	67.3	0.07
Smoking (%)										
Never smoker	39.4	39.1	40.9	40.9	42.2	41.6	42.3	43.4	43.9	0.01
Previous smoker	37.3	37.9	39.4	40.0	38.8	39.3	40.4	39.6	39.5	0.01
Current smoker	18.8	19.6	19.7	19.1	18.9	19.1	17.3	17.0	16.5	0.07
Hyperlipidemia (%)	53.5	55.9	56.3	55.8	51.4	49.9	49.7	49.7	50.2	0.07
Previous infarction (%)	30.7	30.1	29.1	28.1	26.6	25.8	25.6	25.2	23.9	0.06
Previous PCI (%)	25.6	25.3	25.1	24.7	23.4	24.2	23.7	24.2	23.4	0.02
Previous CABG (%)	10.5	10.2	9.6	8.6	8.4	7.7	7.3	7.0	7.4	0.09
Time to angiography/PCI (days)	2.9	2.9	2.6	2.6	2.6	2.3	2.3	2.1	2.1	0.03
Killip class										
Killip I	95.5	96.3	96.4	96.9	97.0	96.6	97.2	97.3	97.2	0.12
Killip II	3.2	2.7	2.5	2.1	2.1	2.3	2.0	1.9	1.9	0.09
Killip III	1.1	0.7	0.7	0.6	0.6	0.7	0.7	0.4	0.7	0.04
Killip IV	0.3	0.4	0.5	0.4	0.3	0.4	0.2	0.4	0.2	0.08
Radial artery access (%)	61.3	68.8	74.6	78.5	82.3	84.9	86.0	87.5	87.3	0.11

© 2020 Dworeck C et al. *JAMA Network Open.*

Procedure off-hours (%)	10.9	12.2	15.4	15.6	17.2	18.8	19.2	19.7	21.4	0.07
Arteries with stenosis (%)										
0	2.9	3.2	3.5	3.5	3.4	4.9	5.4	5.1	5.1	0.06
1	44.8	46.1	45.7	46.1	45.4	45.2	43.6	44.3	42.8	0.03
>2 and/or LM	52.2	50.7	50.8	50.3	51.1	49.9	51.0	50.6	52.1	0.01
Complete revascularization (%)	64.4	67.4	67.7	68.5	68.7	70.1	70.1	71.1	67.8	0.08
PCI with stent (%)										0.04
Drug-eluting stent	40.4	51.8	66.1	77.8	81.4	83.1	83.6	83.2	80.7	0.08
Bare metal stent	46.1	34.5	19.2	6.9	4.2	1.6	0.4	0.2	0.2	0.03
No stent	13.5	13.7	14.7	15.3	14.4	15.4	16.1	16.6	19.2	0.07
$P2Y_{12}$ antagonist (%)										
Clopidogrel	95.7	92.4	63.4	35.7	25.0	19.5	16.4	15.8	16.0	0.09
Ticagrelor	0.0	0.5	34.4	63.1	74.3	79.9	83.4	84.0	83.8	0.05
Prasugrel	4.3	7.1	2.2	1.2	0.7	0.6	0.2	0.2	0.2	0.13
Thrombus aspiration %)	4.1	3.6	3.0	2.6	1.4	1.1	1.2	1.0	0.9	0.05
Direct stenting %)	18.9	17.7	17.0	15.3	13.9	13.9	13.8	12.2	10.9	0.02
Bivalirudin (%)	24.0	25.3	22.9	17.5	12.9	13.1	9.9	2.0	0.4	0.02
GP2b/3a inhibitor (%)	6.6	4.4	2.5	2.4	2.0	1.1	1.5	1.3	1.4	0.01
Unfractionated heparin (%)	79.6	80.5	84.0	90.0	92.5	93.1	93.7	95.9	96.5	0.18

Stand.Diff.=standardized difference $P2Y_{12}$ pretreated vs. not pretreated, stratified by year.

© 2020 Dworeck C et al. *JAMA Network Open.*

eFigure 1. Changing Trend in Pretreatment With P2Y12 Receptor Antagonists Before PCI in Patients With NSTE-ACS Between 2010 and 2018 in Sweden

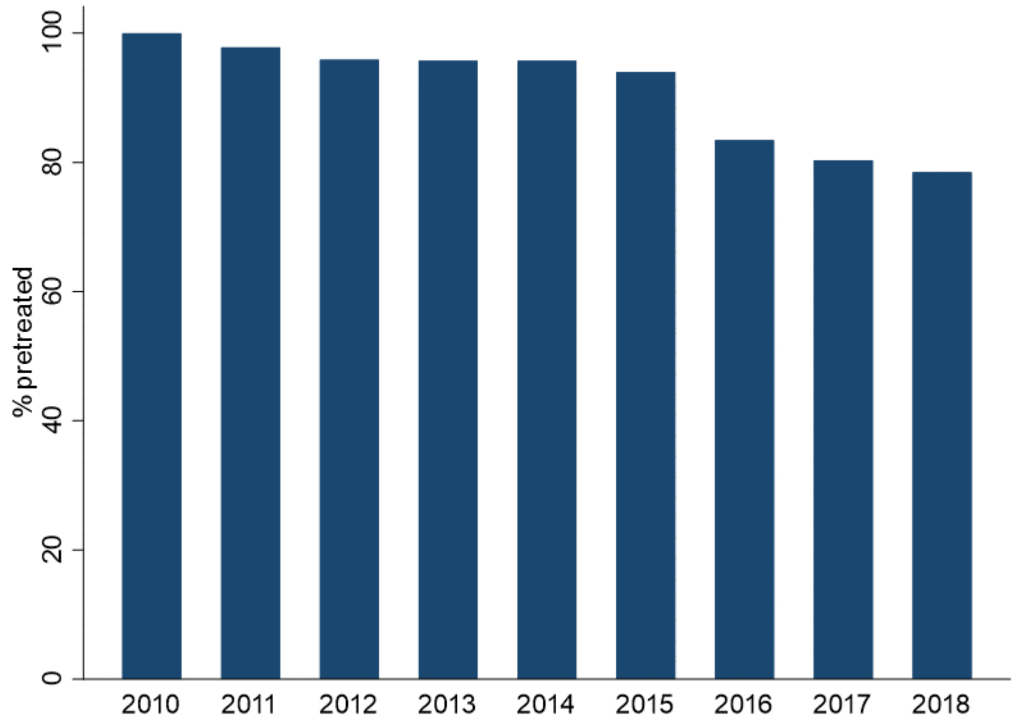

© 2020 Dworeck C et al. *JAMA Network Open.*

eFigure 2. Frequency in Pretreatment With P2Y12 Receptor Antagonists Before and After the Change in the Policy for Routine Pretreatment With P2Y12 Receptor Antagonists Before PCI in Patients With NSTE-ACS in Västra Götaland County

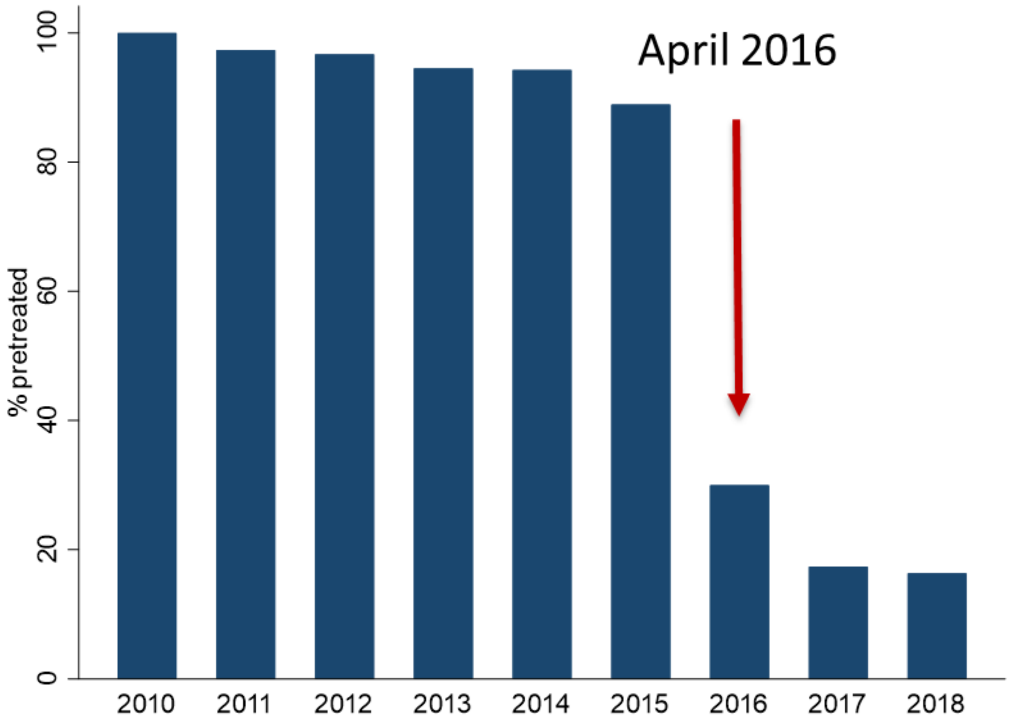

© 2020 Dworeck C et al. *JAMA Network Open.*
